# The association of smart mobile phone usage with cognitive function impairment in Saudi adult population

**DOI:** 10.12669/pjms.36.7.2826

**Published:** 2020

**Authors:** Thamir M. Al-khlaiwi, Syed Shahid Habib, Sultan Ayoub Meo, Mohammed S. Alqhtani, Abeer A. Ogailan

**Affiliations:** 1 Thamir M. Al-Khlaiwi Department of Physiology, College of Medicine, King Saud University, Riyadh, Saudi Arabia; 2 Syed Shahid Habib Department of Physiology, College of Medicine, King Saud University, Riyadh, Saudi Arabia; 3 Sultan Ayoub Meo Department of Physiology, College of Medicine, King Saud University, Riyadh, Saudi Arabia; 4 Mohammed S Alqhtani Medical Student, College of Medicine, King Saud University; 5 Abeer A. Ogailan Independent Researcher

**Keywords:** Mobile phone, Hazards, Radiations, Cognitive function impairment, MOCA

## Abstract

**Background & Objectives:**

Excessive use of mobile phones has raised a great concern about adverse health effects on human health. The present study’s aim was to investigate the association of the usage of smartphones with cognitive function impairment in the Saudi adult population.

**Methods:**

The present cross-sectional study was conducted in the Department of Physiology, College of Medicine, King Saud University, Riyadh, Saudi Arabia during September 2019 to January 2020. A total of 251 Saudi adults who were using mobile phones were recruited, and knowledge, attitude and practices were assessed by interview using a predesigned proforma. The Montreal Cognitive Assessment (MOCA) tool was employed to assess the cognitive functions, comparison was made between daily mobile phone usage group and their correlated Montreal Cognitive Score (MOCA).

**Results:**

The mean age for 251 Saudi adult participants was 32.43 ± 12.8 years. More than 80% of the participants used their mobile phone for more than two hours daily. About 61% of the participants were not aware of the side effect of the radiation generated from mobile phone. The participants showed a decrease in MOCA score with increased daily mobile phone usage (MOCA=26.8 for <1 hour daily usage, 26.1 for 1-2 hours, and 24.6 for >2 hours with P< 0.05). In addition, participants showed decreased MOCA score by keeping their mobile phone near their pillow while sleeping; MOCA=24.35 for near pillow groups and >25.5 for the groups that placed their mobile phone away from the pillow.

**Conclusions:**

Excessive use of mobile phones can cause cognitive function impairment. Strict policies must be implemented to control the use of smartphones in order to minimize the effects on mobile phone radiation on cognition. The media has to be on the forefront in educating the public about the proper usage of mobile phones.

## INTRODUCTION

The smartphones are containing multiple sophisticated features and became an inherent part of human life. It allows users to keep personal information, health and financial data, pictures and memories. Smartphones also became an integral part of modern telecommunications facilities, knowledge and multiple learning options to users, and becomes source of daily activities. The use of smart phone has increased in the last decade, and there is a great debate among people that whether smart phones have facilitated their daily tasks and have made every day life’s needs more convenient.^1^,^2^

The literature shows that mobile phones can negatively affect the human health.^3^,^4^ The World Health Organization (WHO) revealed that exposure to Radiofrequency Electromagnetic Field Radiation (RF-EMFR) generated from mobile phones increases body core temperature and can cause cognition functions impairment.^5^ The children who are exposed to RF-EMF radiation exhibit decreased motor skills as well as attention span and working memory,^6^ poor attention and concentrations.^7^,^8^ Moreover, literature also acknowledge the adverse effects of smartphone.^9^-^13^

Although limited research has been conducted concerning the potential cognitive impacts of smartphone use in the Saudi society, the present study aim was to determine the association of use of smart mobile phone with cognitive function impairment in Saudi adult population.

## METHODS

The present cross-sectional study was conducted in the Department of Physiology, College of Medicine, King Saud University, Riyadh, Saudi Arabia during September 2019 to January 2020. A total of 251 Saudi adult volunteers aged from 15-65 years using mobile phones were invited to participate in this study. Knowledge, attitude and practices were assessed by interview using a specially designed questionnaire in Arabic or English languages. Three questions were designed to determine the awareness of participants about mobile phone hazards. The Montreal Cognitive Assessment (MOCA) was used to assess cognition. From all the participants, a written consent was obtained who voluntarily agreed to join the research project, where they have the opportunity to read the research objectives and join or withdraw from the research at any time, without any profits or penalties. Subjects were recruited through convenience sampling technique. MOCA scores range between 0 and 30, score of 26 or over was considered normal, lower scores <26 indicate mild cognitive impairment (MCI).^14^

### Selection Criteria

Mobile phone users with history of known psychiatric disorders, central nervous system disorders, living near high voltage electricity towers, and subjects with chronic debilitating disorder such as diabetes mellitus, cardiac failure, malignancy were not included in the study. Participants who were below 15 or above 65 were excluded. Mobile phone users who smoke cigarette, shisha were also excluded. One investigator interviewed 251 volunteer male and female mobile phone users and a detailed clinical history was obtained, participant demographic and other characteristics were obtained (Table-I and II).

**Table-I:** Demographic characteristics of all participants and MOCA Scores (n=251).

Characteristics of participants	Mean	SD	Minimum	Maximum
Age	32.43	12.80	15	65
Starting age for use	19.71	9.16	7	53
Age at using reading glasses	32.73	14.95		
*Educational Level: n(%)*				
Secondary	97 (38.6)			
Bachelor	134 (53.4)			
Higher	20 (8.0)			
*Type of mobile phone: n(%)*				
Iphone	159 (63.3)			
Samsung	45 (17.9)			
Huawei	44 (17.5)			
Other	3 (1.2)			
*Years of usage: n(%)*			
1-5	36(14.3)			
6-10	60(23.9)			
>10	154(61.4)			
MOCA	25.02	2.49	17	30

MOCA: Montreal cognition assessment. Values are expressed in mean and standard deviation.

**Table-II:** Attitude and practices of mobile phone users.

Attitude of mobile phone users	Categories	Number (%)
Daily usage	<1 hour	12(4.8)*
	1-2 hour	36(14.3)
	>2hours	203(80.9)
How do you use your mobile?	Handheld	155(61.8)*
	Earphone	47(18.7)
	Speaker	39(15.5)
	Bluetooth	10(4.0)
Do you live near mobile tower	Yes	46(18.3)*
	No	205(81.7)
Do you live near high voltage tower?	Yes	
	No	251(100)
Do you use reading glasses?	Yes	62(24.7)*
	No	189(75.3)
Where do you put your mobile while sleeping?	Near pillow	136(54.2)*
	Inside bedroom	99(39.4)
	Outside bedroom	16(6.4)
What is your dominant hand?	Right	228(90.8)*
	Left	23(9.2)
Where do you put the mobile while calling?	Right ear	178(70.9)*
	Left ear	39(15.5)
	Variable	34(13.5)
Where do you put the mobile when not used?	Upper pocket	8(3.2)*
	Lower pocket	126(50.2)
	Away from pocket	66(26.3)
	Variable	51(20.3)
Do you think you are dependent on mobile?	Yes	141(56.2)*
	To some extent	81(32.3)
	No	29(11.6)
Do you want to quit using mobile?	Yes	30(12)*
	To some extent	62(24.7)
	No	159(63.3)

* ANOVA comparison P <0.001

### Ethics Statement

This study was executed in harmony with the “Declaration of Helsinki,” and the protocol was approved by the “Institutional Review Board, Ethics Committee,” College of Medicine Research Centre, King Saud University (E-19-4232).

### Statistical Analysis

The data were entered into the computer, SPSS V. 22 and Microsoft Windows was used. Continuous variables were expressed as the mean ± standard deviation and descriptive data were expressed as percentages (%). Variables were compared between different groups by one-way ANOVA regarding all variables of knowledge, attitude and different practices of smart mobile phone users. Proportions were compared between different groups using Chi-square test. A p value <0.05 was considered significant.

## RESULTS

More than 61% of the participants used the mobile phone for more than 10 years Table-I. More than 80% of the participants used the mobile phone for more than two hours daily. Table-II. In addition, more than 61% of participants used handheld mode for calling rather than placing the mobile phone away from body in order to minimize the radiation effect (Table-III). About 54% of the participants placed their mobile phone near their heads while sleeping. About 61% of the participants were not aware of the side effect of the radiation of the mobile phone.

**Table-III:** Awareness of mobile phone side effects in all participants

Awareness of the side effect of mobile	Categories	Number (%)
Aware of putting the mobile 5 CM away from your body reduces the radiation effect four times	Do not know	154 (61.4)
	To some extent	47 (18.7)
	Know	50 (19.9)
WHO announcement (mobile phone is a possible cause of cancer)	Do not know	158 (62.9)
	To some extent	59 (23.5)
	Know	34 (13.5)
ACS announcement (mobile phone is a possible cause of brain cancer)	Do not know	173 (68.9)
	To some extent	50 (19.9)
	Know	28 (11.2)

WHO: world health organization. ACS: American cancer society.

**Fig.1:**
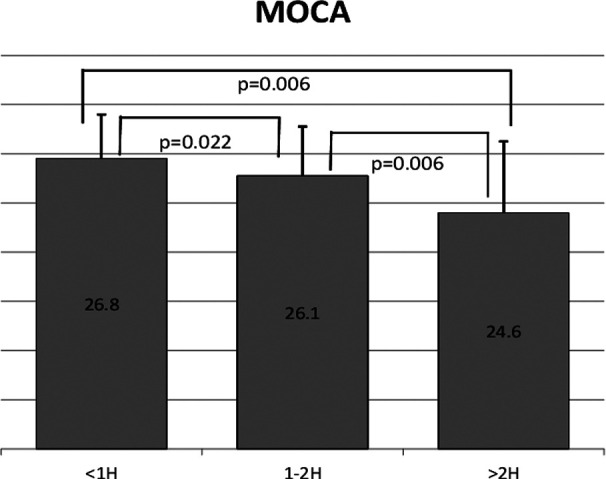
Comparison of MOCA scores between different durations of daily mobile phone usage.

The participants who exceeded two hours of daily usage, in them MOCA score decreased below normal (Fig.1). It also showed a significant difference between groups in the MOCA score except between those who used the mobile less than one hour and two hours.

**Fig.2:**
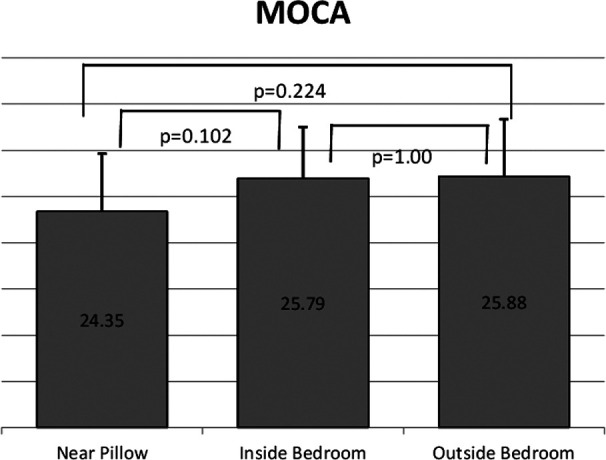
Comparison of MOCA scores between different placement of mobile phone while sleeping.

The association of placement of the mobile phone while sleeping on cognition scores was also assessed (Fig.2). It was noticed that MOCA score was higher while users placed the mobile phone away while sleeping. Even though the effect of the placement of the mobile phone during sleep did not bring the scores to the normal level, but there was a significant effect on the scores. Fig.2 also shows significant difference between the effect of the placement of the mobile phone near the pillow and placing it inside the bedroom or outside on the MOCA score, but there was no significant difference between placing the mobile phone near the pillow and inside the bedroom versus outside the bedroom. However, there was no effect of the placement of the mobile phone while not calling on the scores (Table-IV).

**Table-IV:** MOCA scores and the placement of mobile while not calling.

			Mean	Std. Deviation
MOCA	Upper Pocket	8(3.2)*	23.38	8.297
	Lower Pocket	126(50.2)	25.01	2.560
	Away	66(26.3)	24.88	2.527
	Variable	51(20.3)	25.08	2.440

	Total		24.94	2.853

## DISCUSSION

Exposure to Radiofrequency Electromagnetic Field Radiation (RF-EMFR) has various effects on human health including fatigue, headache, tension, sleep disturbance,^15^ hearing and vision complaints^16^ and risk of type 2 diabetes mellitus.^17^ Extensive fixing of mobile phone base station towers (MPBSTs) in densely populated commercial, residential areas, and school buildings has started community concerns about adverse effects on human health,^6^ mainly on brain functions.^18^

To the best of our knowledge, this is the first study that investigate the relation between the knowledge, attitude and practices of mobile phone usage with cognitive impairment in Saudi adult population. In this study, it was identified that the cognitive functions were deteriorated with the increase daily usage of mobile phone. The present study results are in line with the results of other studies published in different countries. In the present study, the deterioration of cognitive function due to mobile usage was consistent with the conclusion of earlier published literature.^19^

Arns et al.^20^ reported that decrease in brain activity was associated with the use of mobile phone 22. In addition, Kalafatak et al.^8^ found that mobile phone usage has a significant negative impact on working memory performance. The effect was noticed even after the 5-minutes use of mobile phone.

It has also been reported that continuous using or checking of the smart phone screen was associated with cognitive function impairment. The present study has explored how performance was affected among individuals who use their phones to take a break from job tasks. The data showed that individuals who took break on their phones have shown cognition decline which was evident on their weak performance.^21^ Moreover, sleep disorders have been associated with excessive use of smart phones.^22^ Consequently, less sleep duration can lead to impairments in cognitions functions. In addition to the daily usage, deterioration of cognition was associated with placement of mobile phone while sleeping and subsequently cognitive deterioration. The present study results are in consistent with other studies which concluded that mobile phones can affect cognition functions as a result of sleep disturbance.^23^,^24^

The general population need to be educated and counselled on the proper usage and practices of mobile phones. They need to be compelled to ensure mobile phone usage only for essential tasks and should be organized so it will not affect the performance of the students and the employees. Paul et al.^25^ suggested that it is highly needed to teach people to be educated and structured, to know when to have the cell phone on, and to avoid becoming the slave of technology instead of its mastery. In addition, media has to come to frontline to educate the public about the side effects of the improper usage of such device.

### Strengths of the study

This is the first study to investigate the effect of mobile phone usage on cognition functions in Saudi adult population. The study exclusion criteria were highly standardized.

### Limitations of the study

It includes its cross-sectional design and small number of sample size. It was very difficult to find participants who use mobile phone less than one hour daily which reflected that it has become a known habit in the society. The reason to find more participants who used mobile phone for less than two hours was the attempt to increase the sample size. The difficulties to find non-smoking participants, without any debilitating disorders or central nervous systems disorders, and not living near high voltage electricity towers to eliminates the effects of these factors on the results should be countered. Further studies with larger sample size are essential to confirm the current evidence of the role of mobile related disturbance to cognition.

## CONCLUSIONS

Excessive use of mobile phone is associated with cognitive function impairment assessed by Montreal cognitive Assessment (MOCA) score. The health authorities should employ strict policies regarding mobile phones in order to minimize their hazardous effects on human health including cognitive functions impairment. The media has to be on the forefront in educating the public about the proper usage of mobile phone.

### Authors’ Contribution:

**TA, SS** conceived, designed, statistical analysis, editing of manuscript, accuracy and responsivity of the work.

**MA, AO** data collection. **SAM critical review and manuscript editing.**

## References

[1] Alhazmi AA, Alzahrani S, Baig M, Salawati E, Alkatheri A (2018). Prevalence and factors associated with smartphone addiction among medical students at King Abdulaziz University, Jeddah. Pak J Med Sci.

[2] Alosaimi FD, Alyahya H, Alshahwan H, Al Mahyijari N, Shaik SA (2016). Smartphone addiction among university students in Riyadh, Saudi Arabia. Saudi Med J.

[3] Kim JH, Lee JK, Kim HG, Kim KB, Kim HR (2019). Possible Effects of Radiofrequency Electromagnetic Field Exposure on Central Nerve System. Biomol Ther (Seoul).

[4] Meo S, Arif M, Rashied S, Khan MM, Vohra MS, Usmani A, Muhammad Babar Imran Al-Drees AM (2011). Hypospermatogenesis and spermatozoa maturation arrest in rats induced by mobile phone radiation. J Coll Physicians Surg Pak.

[5] Beaglehole R, Bonita R, Kjellstrom T (1993). Basic Epidemiology. World Health Organization (WHO). WHO report:chapter 7, Switzerland, Geneva.

[6] Meo SA, Almahmoud M, Alsultan Q, Alotaibi N, Alnajashi I, Hajjar WM (2018). Mobile Phone Base Station Tower Settings Adjacent to School Buildings:Impact on Students'Cognitive Health. Am J Men's Health.

[7] Deniz O, Kaplan S, Selcuk M, Terzi M, Altun G, Yurt K (2017). Effects of short and long term electromagnetic fields exposure on the human hippocampus. J Microsc Ultrastruc.

[8] Kalafatak F, Bekiaridis-moschou D, Gkioka E, Tsolaki M (2017). Mobile phone use for 5 minutes can cause significant memory impairment in humans. Hellenic J Nuclear Med.

[9] He J, Tu Z, Xiao L, Su T, Tang Y (2020). Effect of restricting bedtime mobile phone use on sleep, arousal, mood, and working memory:A randomized pilot trial. PLoS One.

[10] Schoeni A, Roser K, Roosli M (2015). Memory performance, wireless communication and exposure to radiofrequency electromagnetic fields:a prospective cohort study in adolescents. Environ Int.

[11] Foerster M, Thielens A, Joseph W, Eeftens M, Roosli M (2018). A prospective cohort study of adolescents'memory performance and individual brain dose of microwave radiation from wireless communication. Environ Health Perspect.

[12] Deshmukh PS, Nasare N, Megha K, Banerjee BD, Ahmed RS, Singh D (2015). Cognitive Impairment and Neurogenotoxic Effects in Rats Exposed to Low-Intensity Microwave Radiation. Int J Toxicol.

[13] Forouharmajd F, Ebrahimi H, Pourabdian S (2018). Mobile Phone distance from head and temperature changes of radio frequency waves on brain tissue. Int J Prev Med.

[14] Milani SA, Marsiske M, Cottler LB, Chen X, Striley CW (2018). Optimal cutoffs for the Montreal Cognitive Assessment vary by race and ethnicity. Alzheimers Dement (Amst).

[15] Al-Khlaiwi T, Meo SA (2004). Association of mobile phone radiation with fatigue, headache, dizziness, tension and sleep disturbance in Saudi population. Saudi Med J.

[16] Meo SA, Al-Drees AM (2005). Mobile phone related hazards and subjective hearing and vision symptoms in the Saudi population. Int J Occupat Med Env Health.

[17] Meo SA, Alsubaie Y, Almubarak Z, Almutawa H, AlQasem Y, Hasanato RM (2015). Association of exposure to Radio-Frequency Electromagnetic Field Radiation (RF-EMFR) generated by mobile phone base stations with Glycated Hemoglobin (HbA1c) and risk of type 2 diabetes mellitus. Int J Env Res Pub Health.

[18] Saikhedkar N, Bhatnagar M, Jain A, Sukhwal P, Sharma C, Jaiswal N (2014). Effects of mobile phone radiation (900 MHz radiofrequency) on structure and functions of rat brain. Neurolog Res.

[19] Extremera N, Quintana-Orts C, Sanchez-Alvarez N, Rey L (2019). The role of cognitive emotion regulation strategies on problematic smartphone use:Comparison between problematic and non-problematic adolescent users. Int J Environ Res Public Health.

[20] Arns M, Van Luijtelaar G, Sumich A, Hamilton R, Gordon E (2007). Electroencephalographic, personality, and executive function measures associated with frequent mobile phone use. Int J Neurosci.

[21] Kang S, Kurtzberg TR (2019). Reach for your cell phone at your own risk:The cognitive costs of media choice for breaks. J Behav Addic.

[22] Sahin S, Ozdemir K, Unsal A, Temiz N (2013). Evaluation of mobile phone addiction level and sleep quality in university students. Pak J Med Sci.

[23] Dewi RK, Efendi F, Has EMM, Gunawan J (2018). Adolescents'smartphone use at night, sleep disturbance and depressive symptoms. Int J Adolescent Med Health 2018.

[24] Ferguson SA, Appleton SL, Reynolds AC, Gill TK, Taylor AW, McEvoy RD Making errors at work due to sleepiness or sleep problems is not confined to non-standard work hours:results of the 2016 Sleep Health Foundation national survey. Chronobiol Int.

[25] Paul B, Saha I, Kumar S, Samim Ferdows SK, Ghose G (2015). Mobile phones:time to rethink and limit usage. Indian J Public Health.

